# Ewing Sarcoma Cells Secrete *EWS/Fli-1* Fusion mRNA via Microvesicles

**DOI:** 10.1371/journal.pone.0077416

**Published:** 2013-10-04

**Authors:** Masanori Tsugita, Nami Yamada, Shunsuke Noguchi, Kazunari Yamada, Hiroshi Moritake, Katsuji Shimizu, Yukihiro Akao, Takatoshi Ohno

**Affiliations:** 1 Department of Orthopaedic Surgery, Gifu University Graduate School of Medicine, Gifu, Gifu, Japan; 2 United Graduate School of Drug Discovery and Medical Information Sciences, Gifu, Gifu, Japan; 3 United Graduate School of Veterinary Sciences, Gifu University, Gifu, Gifu, Japan; 4 Division of Pediatrics, Department of Reproductive and Developmental Medicine, Faculty of Medicine, University of Miyazaki, Miyazaki, Miyazaki, Japan; University Hospital of Modena and Reggio Emilia, Italy

## Abstract

Tumours defined as Ewing sarcoma (ES) constitute a group of highly malignant neoplasms that most often affect children and young adults in the first 2 decades of life. The *EWS/Fli-1* fusion gene, a product of the translocation t(11;22) (q24; 12), is detected in 95% of ES patients. Recently, it was validated that cells emit a heterogeneous mixture of vesicular, organelle-like structures (microvesicles, MVs) into their surroundings including blood and body fluids, and that these MVs contain a selected set of tumor-related proteins and high levels of mRNAs and miRNAs. In this present study, we detected the Ewing sarcoma-specific *EWS/Fli-1* mRNA in MVs from the culture medium of ES cell lines carrying t(11;22) (q24; 12). Also, we detected this fusion gene in approximately 40% of the blood samples from mice inoculated with xenografts of TC135 or A673 cells. These findings indicate the *EWS/Fli-1* mRNA in MVs might be a new non-invasive diagnostic marker for specific cases of Ewing sarcoma.

## Introduction

Multi-disciplinary care incorporating advances in diagnosis, surgery, chemotherapy, and radiation has substantially improved the survival rate of patients with localized Ewing sarcoma to nearly 70%. However, these advances have not significantly changed the long-term outcome for those individuals with metastatic or recurrent disease, i.e., the 5-year survival remains less than 25% [1]. So, early diagnosis and follow-up aided by a novel prognostic biomarker would be desirable [2]. ES cells have the t (11,22)(q24;q12) translocation which results in the formation of *EWS/Fli-1* fusion genes. The origin of ES and the relationship of EWS/FLI-1 has received much debate [3]. The 2 main types of *EWS/Fli-1* fusions, a fusion of *EWS* exon 7 to *FLI1* exon 6 (type 1) and that of *EWS* exon 7 to *FLI1* exon 5 (type 2), account for about 60 and 25% of cases, respectively. In our laboratory, we have focused on developing a new treatment strategy for Ewing sarcoma [4–8]. Previously we reported that silencing EWS/Fli-1 by the use of fusion mRNA-specific siRNA strikingly reduces cell proliferation both *in vitro* and *in vivo* [7].

Recently, it was validated that these cells secrete microvesicles (MVs) into their surrounding body fluids and blood, with MV 30-1000 nm in diameter containing genetic products such as mRNA, miRNA and protein. Vesiculation events occur either at the plasma membrane (shedding microvesicles [SMVs]) or within endosomal structures (exosomes [EXOs]). These MVs contain growth factors and their receptors, proteases, adhesion molecules, and signaling molecules, as well as DNA, mRNA, and microRNA (miRNA) [9]. More recently, it has been shown that MVs released from tumor cells into the bloodstream of cancer patients contain a selected set of tumor-related proteins and high levels of mRNA and miRNA, molecules that are considered to be communication tools [10]. Interest in using such molecules for diagnosis and treatment has been growing. In this study, we examined whether MVs generated from Ewing sarcoma cells might carry the *EWS/Fli-1* fusion mRNA and found, by using both *in vitro* and *in vivo* systems, that MVs can indeed contained the Ewing sarcoma-specific *EWS/Fli-1* mRNA.

## Methods

### Cell culture

TC135, A673 and SK-ES-1 are ES cell lines carrying the *EWS/Fli-1* gene. TC-135 and A673 produce the *EWS/Fli-1* Type 1 fusion, whereas SK-ES-1 has the Type 2 fusion. TC-135 cells were kindly supplied by Dr. T.J. Triche (University of Southern California, Los Angeles, CA) [11]. MP-CCS-SY is a clear cell sarcoma cell line carrying the *EWS/ATF-1* fusion gene, a product of the translocation t(12;22)(p13;q12). MP-CCS-SY cells were kindly supplied by Dr. Hiroshi Moritake (Division of Pediatrics, Department of Reproductive and Developmental Medicine, Faculty of Medicine, University of Miyazaki, Japan) [12]. HOS is an osteosarcoma cell line having no fusion gene. A673, SK-ES-1 and HOS cells were purchased from the American Type Culture Collection (Manassas, VA). All cells were cultured at 37°C under a 5% humidified CO2 atmosphere. TC135 and MP-CCS-SY cells were maintained in RPMI1640 medium (Invitrogen, Carlsbad, CA) containing 5% fetal bovine serum (FBS). A673 and HOS cells were cultured in DMEM (Wako, Osaka, Japan) with 10% FBS. SK-ES-1 cells were cultured in McCoy’s 5A medium (Invitrogen, Carlsbad, CA) with 10% FBS.

### Isolation of extracellular microvesicles

**Figure 1:**
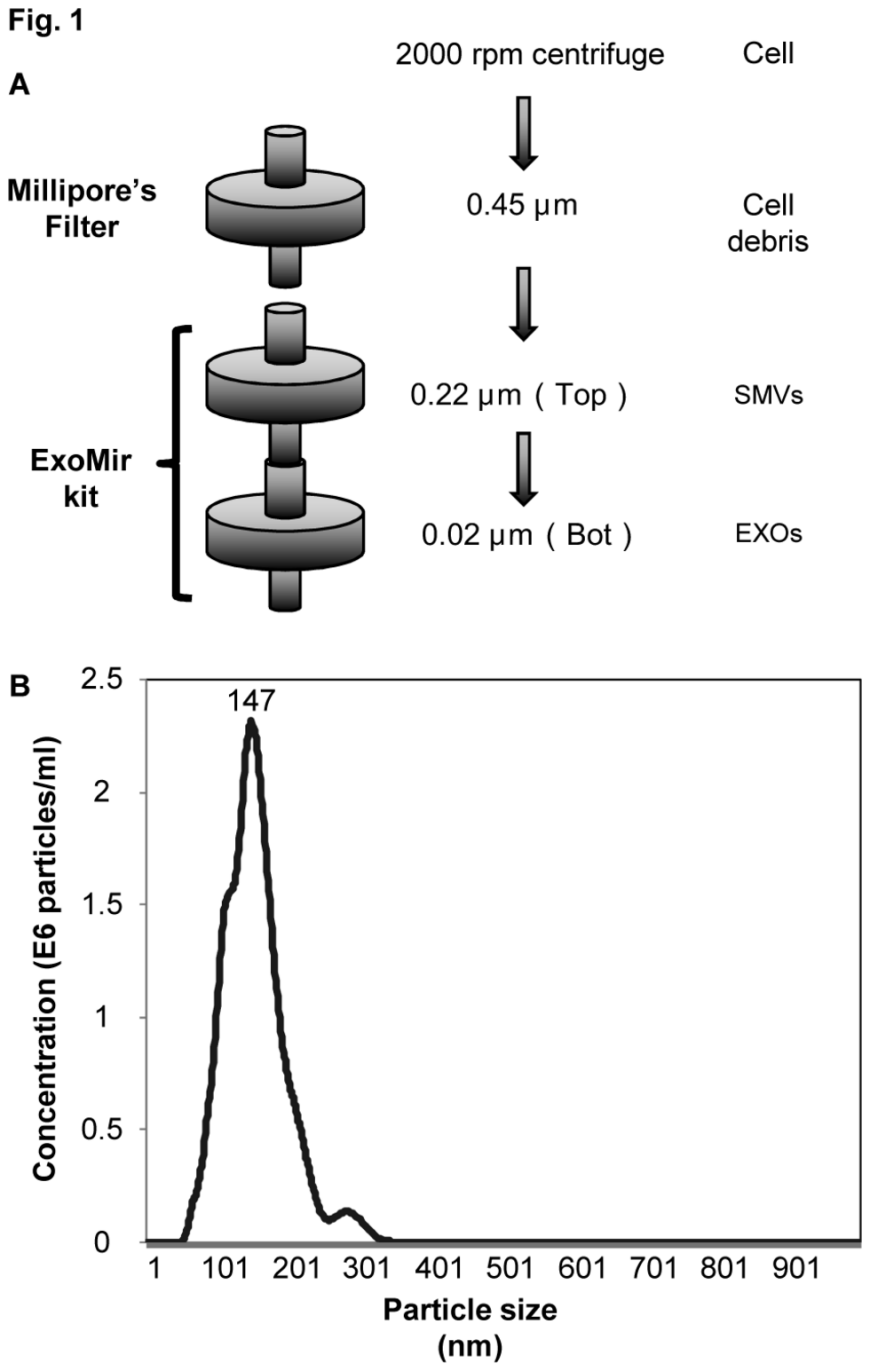
Isolation of microvesicles (MV) and measurement of particle diameter. (A) Isolation of MVs by centrifugation and filtration using the ExoMir kit. For preparation of MVs shed from tumor cells, the medium of the cell cultures was centrifuged at 2000 rpm for 10 min to remove cells and their debris. The supernatant was then sequentially filtered (0.45 µm). The MV samples after passage through the top filter (0.22 µm) of the ExoMir kit were used for Nanoparticle tracking analysis (NTA). SMVs, shedding microvesicles: EXOs, exosomes. "Bot" refers to "Bottom fraction" derived MVs. (B) The Nanosight LM10 nanoparticle characterization system (NanoSight, NanoSight Ltd, UK) equipped with blue-laser (638 nm) illumination was used for real-time characterization of the vesicles. The results are presented at the average value of 2 independent experiments. The number of MVs (E6 particles/ml) and the size distribution (particle diameter, nm) are shown on the *y* axis and *x* axis, respectively.

TC135, A673, SK-ES-1, MP-CCS-SY and HOS cells were grown in medium containing 5% FBS. To exclude cell debris, we subjected the culture medium sequentially to centrifugation at 2000 rpm for 10 min and filtration through a 0.45-µm filter. Then, the supernatant was further filtered through the 2 filters of an ExoMir kit. We obtained the large MVs (mainly SMVs), which passed through the 0.45-µm filter but not the top one (Top) and small MVs (mainly EXOs), which passed through the Top filter but not the Bottom one, supplied in the ExoMir kit (Figure 1A). Also see Movie S1. The total RNA was extracted from the trapped MVs by Top filter (Top fraction) and Bottom one (Bot fraction). We avoid forced filtering so as not to collect broken fragments of MVs.

To verify that we detected EWS/Fli-1 mRNA from MVs and not from contaminating cellular debris, we performed ribonuclease (RNase) treatment before filtration using the ExoMir kit. For this treatment, 2 µl of RNase A (Sigma-Aldrich Co. LLC.) was added to 6 ml of the MV-enrich medium from TC135 cell cultures (final concentration of 8 ng/µl); and then incubated for 30 min. Then 15 µl of Proteinase K was added, followed by incubation for 30 min to minimize the influence of the RNase used. The detection of EWS/Fli-1 mRNA was performed after filtration using the ExoMir kit.

### PCR

**Figure 2:**
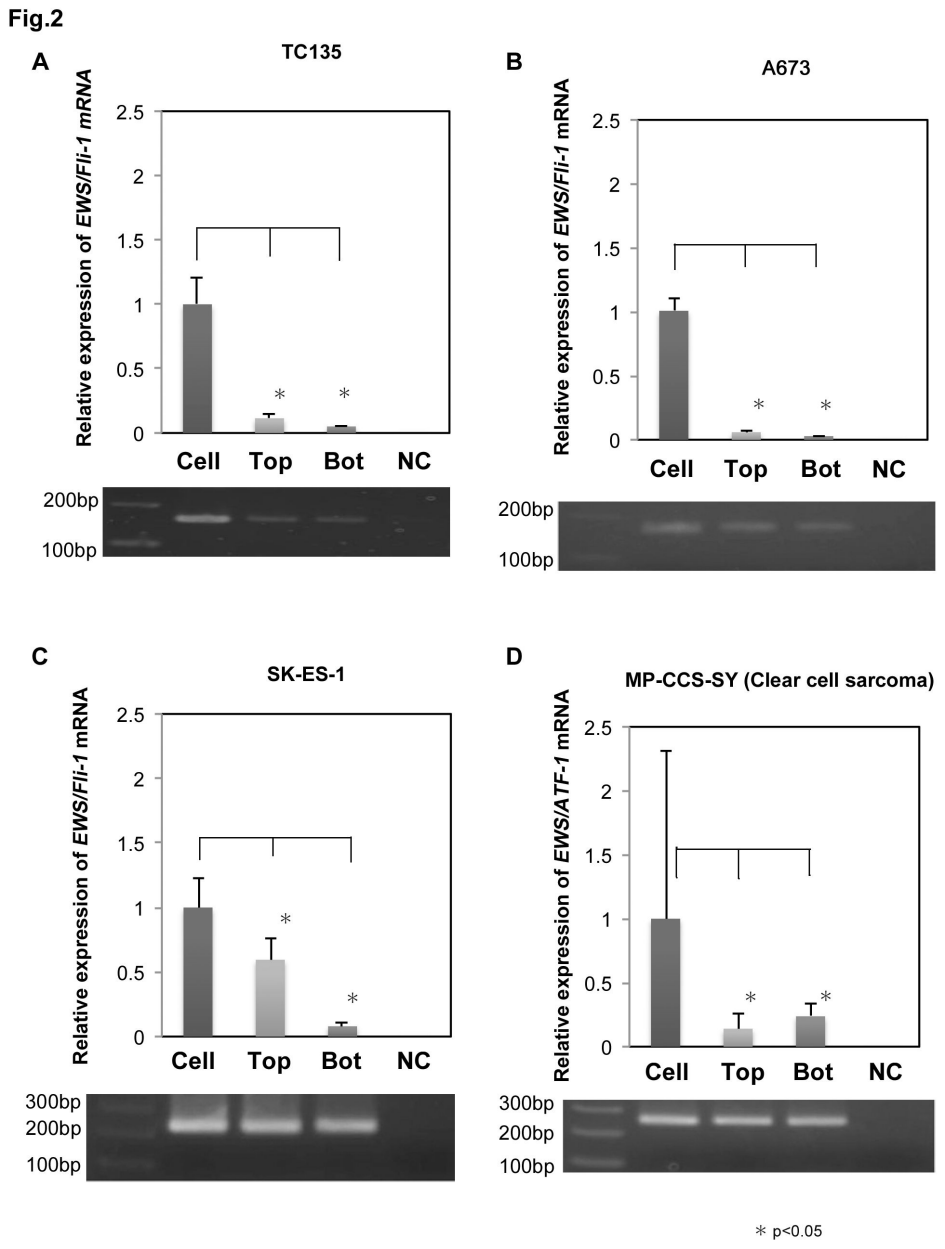
Detection of fusion mRNAs in MVs from ES cells and clear cell sarcoma cells. The levels of fusion mRNAs in the cells and the MV-fractions trapped by Top and Bottom filters of the ExoMir kit. The upper panel shows the relative expression of *EWS/Fli-1* mRNA, the relative quantities are given with the amount in “Cell” indicated as “1”. The films beneath the graphs show the results of the electrophoretic analysis of the PCR products. "Bot" refers to "Bottom fraction" derived MVs. (A, B) TC135 and A673 cells have the type-1 *EWS/Fli-1* fusion. The appropriate amplicon size is 158bp. (C) SK-ES-1 has the type-2 *EWS/Fli-1* fusion gene. The appropriate amplicon size is 224bp. (D) MP-CCS-SY is a clear cell sarcoma cell line which has *EWS/ATF-1* fusion gene. The appropriate amplicon size is 246bp.

**Figure 3:**
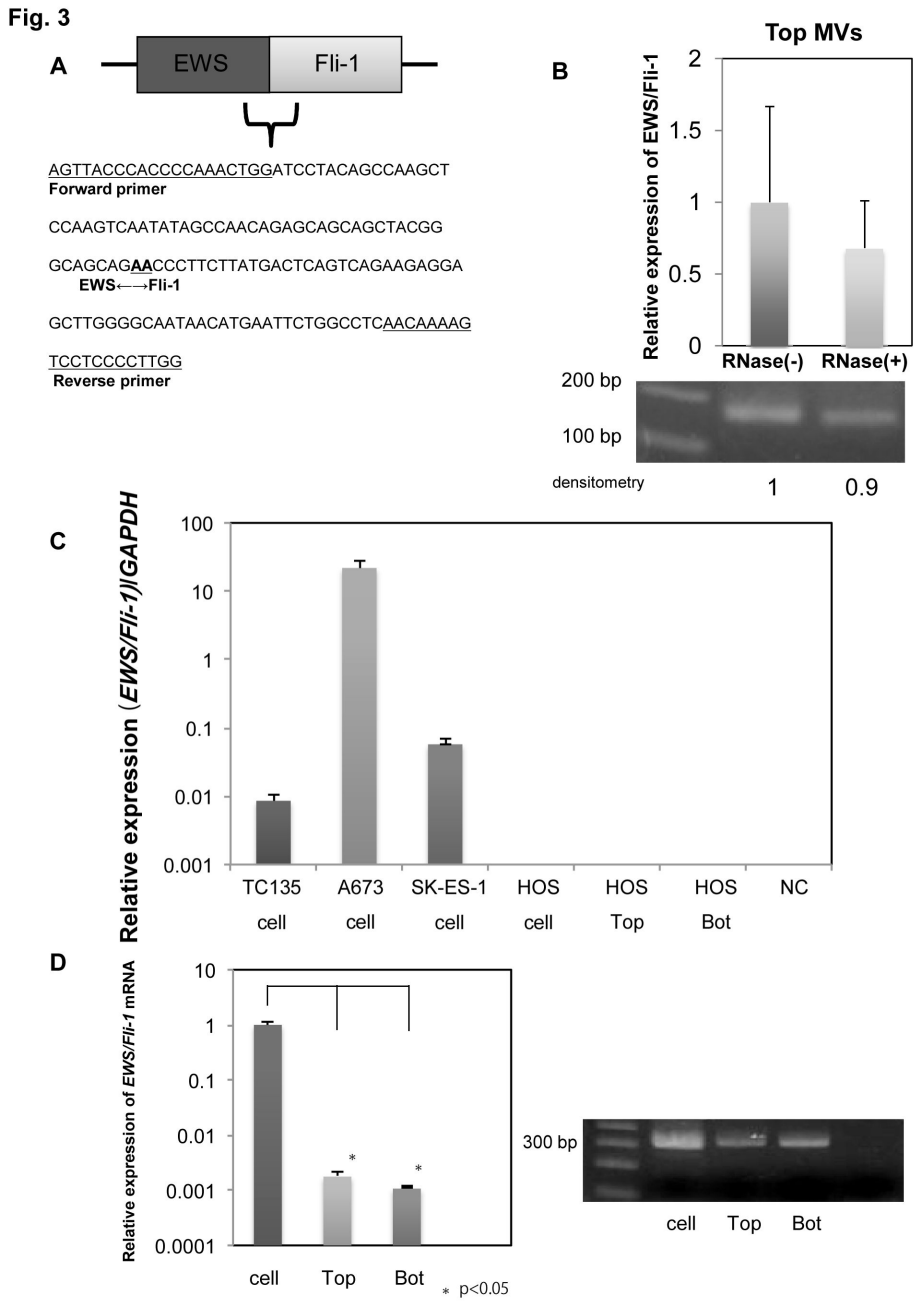
Validation of the presence of the EWS/Fli-1 fusion mRNAs in MVs from TC135 cells. (A) Nucleotide sequence of the PCR products obtained from the agarose gel shown in “Figure 2A”). The sequence is that of the *EWS/Fli-1* fusion mRNA, and the breakpoint of the *EWS/Fli-1* fusion mRNA is indicated. (B) Level of *EWS/Fli-1* mRNA in the Top fraction treated or not with RNase. The levels of the mRNA were estimated by qRT-PCR in upper graph (P=0.47) and electrophoretic analysis in lower film. The quantities in the graph are given relative to the amount for RNase-free treatment indicated as “1.” Quantification of the PCR band was performed, and the results were analyzed with ImageJ (NIH) software. (C) Expression levels of *EWS/Fli-1* mRNA in *EWS/Fli*-1-positive ES cell lines and in the -negative HOS cell line. No PCR product was obtained for the HOS cell line which lacks the fusion mRNA by using the primers indicated in “Figure 2A” for *EWS/Fli-1* fusion mRNAs. (D) The levels of fusion mRNAs in the cells and the MV-fractions trapped by the ExoMir kit were examined by using the other primer set for *EWS/Fli-1* mRNA to check whether we could obtaine the same results with both primer sets. The film at the right shows electrophoretogram of the PCR products, Amplicons of appropriately 321 bp are seen. "Bot" refers to "Bottom fraction" derived MVs.

Total RNA was extracted from the cells and MVs by using an ExoMir kit (Bioo Scientific Corp. USA). One µg of total RNA was reverse-transcribed by using a PrimeScript^tm^ RT reagent kit (TAKARA BIO TECHNOLOGY, Japan). PCR analysis by use of Takara Bio TaqTM (TAKARA BIO TECHNOLOGY, Japan) was performed in accordance with the manufacturer’s instructions. Quantitative real-time PCR analysis using the fluorescent SYBR green method (THUNDERBIRD ^tm^SYBRqPCR Mix; TOYOBO CO, LTD Japan) was performed in accordance with the manufacturer’s instructions. The primers 5’-AGTTACCCACCCCAAACTGG-3’ (forward) and 5’-CCAAGGGGAGGACTTTTGTT-3’ (reverse) were used to amplify *EWS/Fli-1* mRNA (Figure 2A-C) [7], the primers 5’-AGCAGTTACTCTCAGCAGAACACC-3’ (forward) and 5’-CCAGGATCTGATACGGATCTGGCTG-3’ (reverse) were also used to amplify EWS/Fli-1 mRNA (Figure 3D). *EWS/ATF-1* mRNA was amplified by use of primers 5’-GAGGCATGAGCAGAGGTGG-3’ (forward) and 5’-GAAGTCCCTGTACTCCATCTGTG-3’ (reverse; Figure 2D). Amplification of GAPDH mRNA was achieved with primers 5’-CCACCCATGGCAAATTCCATGGCA-3’ (forward) and 5’-TCTAGACGGCAGGTCAGGTCCACC-3’ (reverse). The real-time PCR program consisted of enzyme activation at 95°C for 30 sec followed by amplification for 40 cycles (95°C for 5 sec, 62°C for 60 sec). Data were generated from each reaction and analyzed for gene expression by using a Thermal Cycler Dice RealTime SystemⅡ(TAKARA BIO TECHNOLOGY, Japan)

### Nanoparticle Tracking Analysis (NTA)

Nanoparticle tracking analysis (NTA) is an innovative system for sizing particles from about 30 to 1,000 nm, with the lower detection limit being dependent on the refractive index of the nanoparticles. This technique combines laser light scattering microscopy with a charge-coupled device (CCD) camera, which enables the visualization and recording of nanoparticles in solution. NTA measurements were performed with a NanoSight LM20 (NanoSight, Amesbury, United Kingdom), equipped with a sample chamber having a 640-nm laser and a Viton fluoroelastomer O-ring. Measurements were performed at room temperature. NTA 2.2 Build 0366 software was used for capturing and analyzing the data. The mean size and SD values obtained by the NTA software correspond to the arithmetic values calculated for the sizes of all the particles analyzed by the software [13,14].

### Tracing of nascent *EWS/Fli-1* mRNAs from TC135 cells into microvesicles by using 5-ethynyl uridine

This experiment was performed by using a Click-iT Nascent RNA Capture Kit (Invitrogen, Carlsbad, CA). The Click-iT Nascent RNA Capture protocol begins with the incubation of TC135 donor cells with an analog of uridine, 5-ethynyl uridine (EU, an alkyne-modified nucleoside), which is efficiently and naturally incorporated into the nascent RNA. Donor TC135 cells were incubated overnight in EU-containing culture medium and then the MVs were purified from the culture medium by ultracentrifugation (100,000 rpm, 3 hours). The MV-concentrated solution was diluted with 1 ml of PBS. This PBS solution containing MVs was added to the medium of the recipient cells (TC135, A673, HOS); and, as a negative control, we used another PBS solution without MVs. All recipient cells were then incubated overnight. After the incubation, total RNA was extracted from the cells and MVs and the RNA molecules labeled with EU were used in a copper-catalyzed click reaction with azide-modified biotin, which creates a biotin-based handle for capturing nascent RNA transcripts on streptavidin magnetic beads. The analysis was carried out by performing quantitative RT-PCR (qRT-PCR) as described above.

### Detection of *EWS/Fli-1* mRNA in the plasma samples from ES cell/xenografted mice

Twenty seven female BALB/c Slc-nu/nu nude mice (4 weeks old) were obtained from Japan SLC Inc. (Hamamatsu, Japan). The mice were housed in the animal facilities of the Division of Animal Experiments, Life Science Research Center, Gifu University. A total of 3.0 × 10^6^ TC135 or A673 cells in 0.1 ml of PBS (Wako, Osaka, Japan) were inoculated subcutaneously, through a 26-gauge needle into the posterior flank and hip of 10 mice (5 weeks old). As a control, 10 mice were not inoculated. At 3-4 weeks after the inoculation, the mice were sacrificed; and blood samples (500 µl) were taken from the caudal vena cava. Total RNA from MVs was extracted after trapping the MVs on the filters of the ExoMir kit, as described above. Quantitative real-time PCR analysis was performed to amplify *EWS/Fli-1*, as described for the *in vitro* experiment. Animal experiments in this study were performed in compliance with the guidelines of the Institute for Laboratory Animal Research of Gifu University (approval number: 11021), and with the UKCCCR Guidelines for the Welfare of Animals Used for Experimental Neoplasia.

### Statistical analysis

Statistical analyses were carried out using GraphPad Prism Version 5.01 (GraphPad Software, CA). The data were analyzed using t^2^ test, and differences at P < 0.05 were considered to be significant.

## Results

As shown in Figure 1A, the large MVs, mainly SMVs (100-1000nm), passed through the 0.45-µm filter and were trapped on the Top filter (Top MVs) of the ExoMir kit; and the small ones, mainly EXOs (50-150nm), passed through the Top filter and were trapped on the Bottom filter (Bot MVs). This distribution was confirmed by the findings of nanoparticle tracking analysis (NTA) using NanoSight (Figure 1B). NTA indicated that the diameter of MVs that passed through the Top filter was approximately 147 nm, as shown by the peak in the size-distribution graph (Figure 1B). We confirmed that the Top and Bottom fractions contained mainly SMVs and EXOs, respectively. In order to clarify whether *EWS/Fli-1* fusion mRNAs had been incorporated into the MVs, we examined its levels in MVs from both Top and Bottom fractions. Interestingly, significant EWS/Fli-1 mRNA levels were detected in both fractions in all the Ewing sarcoma cell lines tested (Figure 2A-C). The levels of the fusion mRNAs detected in the Top and Bottom fractions represented almost one-eighth of the intracellular level in TC135 cells. The relative expression of the fusion mRNA with respect to that in the cells was different between the cells and MVs, indicating the different distribution of RNA molecules in the MVs. Electrophoretic analysis of the PCR products indicated the appropriate size of *EWS/Fli-1* PCR products (Figure 2A-C). In MP-CCS-SY (clear cell sarcoma), the *EWS/ATF-1* fusion mRNAs were also detected (Figure 2D), as shown in the Ewing sarcoma cell lines.

The nucleotide sequence of PCR products from TC135 cells was determined and ascertained to be that of *EWS/Fli-1* fusion mRNAs (Figure 3A). In order to verify that we had measured the *EWS/Fli-1* mRNAs in the MVs and not in cellular debris, we further examined the levels of *EWS/Fli-1* mRNA in the Top fraction after RNase treatment. Figure 3B indicates that the level of the fusion mRNA after the treatment was almost maintained compared with that found without the enzyme treatment. Thus, we established a consistent method for isolating MVs by centrifugation and filtration from the samples, and these findings taken together suggest that tumor-specific fusion mRNAs were secreted via MVs from not only Ewing sarcoma cells harboring the *EWS/Fli-1* fusion gene, but also clear cell sarcoma cells harboring *EWS/ATF-1*. The primers used for *EWS/Fli-1* fusion mRNA detected no PCR product in the osteosarcoma cells lacking *EWS/Fli-1* (Figure 3C). We also used another primer set for the *EWS/Fli-1* mRNA, and again detected the fusion mRNA (Figure 3D). Thus, these finding altogether demonstrated that MVs secreted from ES and clear cell sarcoma cells included the *EWS/Fli-1* and *EWS/ATF1* fusion mRNA, respectively. We also examined the surface marker of MVs (CD63) in order to ascertain whether the MVs are concentrated by our method. On the basis of CD63 detection, the concentration of MVs was about 2 times higher than that of cells (Figure S1). The results confirmed that for TC135 cells, CD63 like other tetraspanins can serve as a MV marker [15]. Biochemically, it was demonstrated that our method using Exomir kit concentrated the MVs.

**Figure 4:**
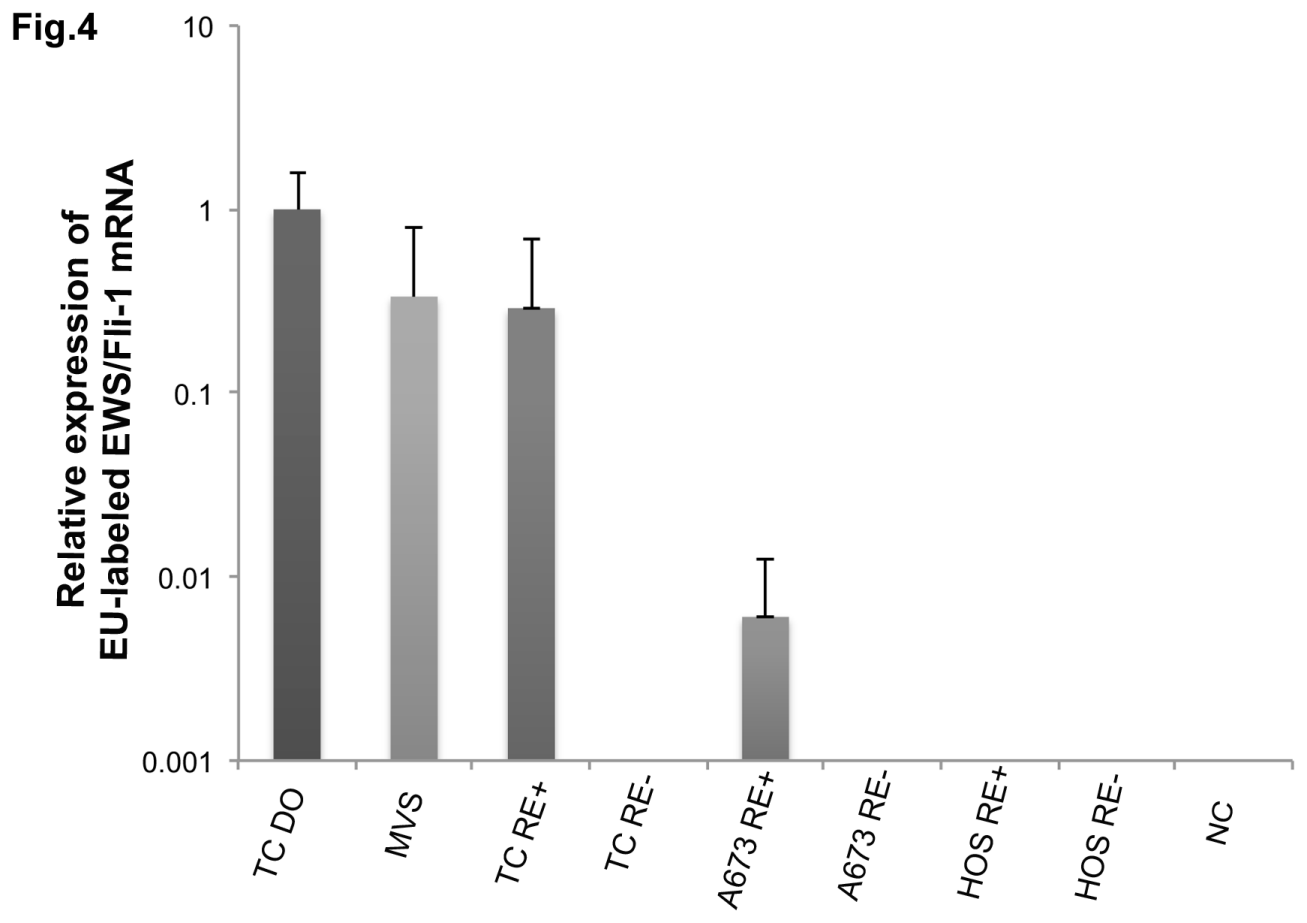
Tracing EU-labeled *EWS/Fli-1* fusion mRNAs from donor cells to recipient cells with transference via MVs. Amounts of EU-labeled *EWS/Fli-1* mRNAs in donor and recipient cells, with transference via MVs, are shown. The relative quantities of EU-labeled *EWS/Fli-1* mRNA are given, with the amount in the expression of TC135 donor cells indicated as “1.”. TC DO: TC135 donor cell, MVs: the microvesicles of TC135 cell, RE+: recipient cells incubated with MVs of TC135 donor cells, RE-: recipient cells without MVs of TC135 donor cells, NC: no-template control.

In order to further validate the secretion of RNA molecules via MVs, we traced the processing of EU-labeled nascent RNA in the TC135 cells. Figure 4 indicates that the intracellular EU-labeled *EWS/Fli-1* mRNAs moved into MVs, as determined by real-time qRT-PCR, and were transferred to TC135 recipient cells. The MVs from TC135 cells were also transferred to other ES cells (A673), but not to non-Ewing sarcoma HOS cells.

**Figure 5:**
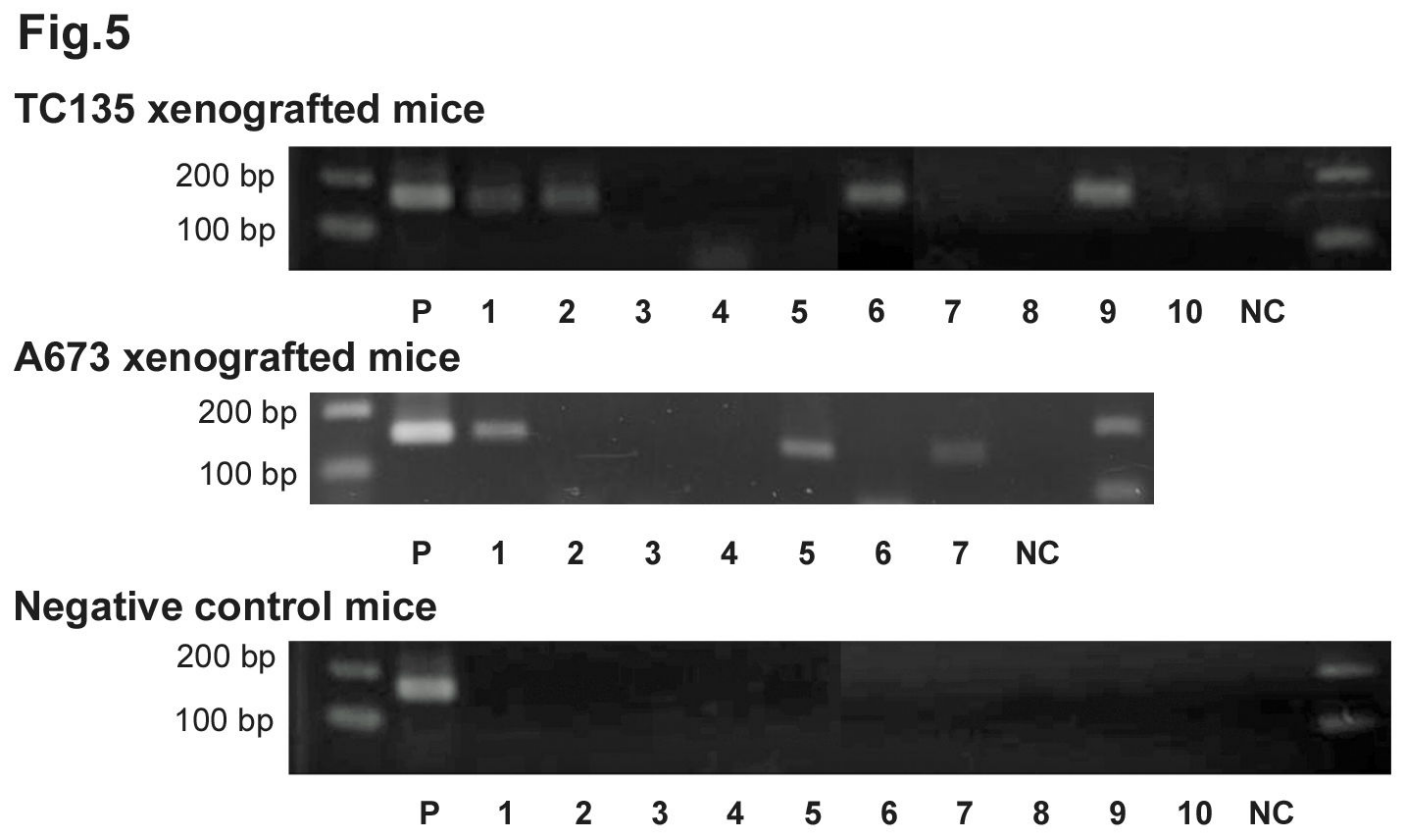
Detection of *EWS/Fli-1* mRNAs in MVs isolated from blood samples taken from ES cell/xenografted mice. The method of isolation of MVs from the blood samples was described in Materials and Methods. Confirmation of the *EWS/Fli-1* mRNAs as the quantitative PCR products was performed. Electrophoretograms of the qRT-PCR products are shown. Among the 10 TC135 tumor-bearing mice, 4 of them were positive for the fusion mRNA. In the case of the 7 A673 tumor-bearing mice, 3 of them were fusion mRNA positive. The positive control from TC135 and A673 cells (P) and non-template control (NC) are also shown.

Next, we examined mice that had been inoculated with EWS/Fli-1 fusion mRNA-bearing ES cells (TC135 or A673) for the presence of this mRNA in blood plasma. Four out of ten mice inoculated with TC135 cells had blood samples that were positive for EWS/Fli-1 PCR products. On the other hand three of the seven mice inoculated with A673 cells had demonstrable EWS/Fli-1 mRNA in the MVs derived from their blood (Figure 5). Thus these in vivo murine models demonstrated that blood-derived MVs could be found in mice bearing TC135 or A673 derived tumors. There seemed to be no relationship between the detection of EWS/Fli1 mRNA in the blood and the tumor volume (data not shown).

## Discussion

MVs are released by various kinds of cells and remain in the extracellular space, such as blood and other biological fluids [16]. Therefore, many studies have been performed to find biomarkers contained in MVs in cancer patients; and the number of MVs in the circulation have been shown to parallel the progression of cancer and poor prognosis [17–19]. Recent studies suggest that MVs induce epigenetic remodeling in the target cells by transferring genetic products [20]. MVs may act as a mediator of cell-to-cell communication, facilitating the exchange of genetic information and MVs in cancer cells may have pleiotropic effects on target cells. Moreover, some genetic products in MVs may serve as biomarkers, such as the recently discovered miRNAs miR-21 and miR-141 [19,21]. However, the biological significance of these findings has not become clear yet. In our laboratory, we have focused on identifying Ewing sarcoma-specific properties to aid in the diagnosis and treatment of this malignant tumor [22–26]. The t(11;22) (q24;q12) translocation is present in up to 95% of cases of Ewing’s sarcoma and results in the formation of an *EWS/Fli-1* fusion gene. Many alternative forms of *EWS/Fli-1* exist because of variations in the locations of the EWS and FLI1 genomic breakpoints. The two main types, fusion of EWS exon 7 to FLI1 exon 6 (type 1) and fusion of EWS exon 7 to FLI1 exon 5 (type 2), account for about 60 and 25%, respectively, of *EWS/FLI-1* fusions. However, alternative EWS-Fli-1 fusion proteins and ES heterogeneity make it at present difficult to define a list of biological biomarkers for practical clinical use [27]. In the current study, we found that the fusion mRNA could be detected in MVs from not only Ewing sarcoma cells (both type 1 and type 2 fusion of *EWS/Fli-1*), but also clear cell sarcoma cells which have another fusion gene (*EWS/ATF-1*). Furthermore, we also found that the fusion mRNA could be detected in MVs in plasma samples from the 2 different ES cell-xenografted mice with TC135 or A673 cells, respectively. These findings suggest the possibility that the *EWS/Fli-1* mRNA of MVs might be useful as a non-invasive diagnostic marker. Balaj et al. reported that microvesicles containing human c-Myc exoRNA could be isolated from serum samples from medulloblastoma-bearing mice and that this exoRNA was detectable in 2/5 (40%) of these mice [9]. This detection rate is almost the same as that of presently obtained. We think our technique is very advantageous, because this marker is completely tumor specific. There are several ways to isolate MVs, including the use of the ExoMir kit [9]. As a method for the isolation of MVs, we established a centrifugation and filtration method. Firstly, the cell debris and apoptotic cells were excluded from the cell culture medium mainly by centrifugation and filtration through a Millipore membrane having 0.45-µm pores. Then, the samples were further processed by sequential filtration through Top (0.22 µm) and Bottom (0.02 µm) filters supplied in the ExoMir kit. The samples thus collected were evaluated for their diameter and number of MVs by performing NTA. Based on size distribution, the major population of the MVs that passed through the Top filter showed a diameter of 147 nm, indicating the nanoparticles to be MVs from TC135 cells. By using the same method, we earlier showed that MVs from prostate cancer cell lines induce the differentiation of pre-osteoblast cells [14]. In this case, the diameters of the MVs estimated by NTA were almost the same as those determined by electron microscopy. The number of MVs collected from 10 ml of culture medium was approximately 1 x 10^9^/ml. The *EWS/Fli-1* mRNA was detected in MVs in both Top and Bottom fractions. The presence of *EWS/Fli-1* in MVs was further confirmed by RNase-treatment experiment (Figure 3B). Moreover, the fusion mRNAs were detected in blood samples from 4 out of 10 and 3 out of 7 mice that had been xenografted with TC135 and A673 cells respectively. There seemed to be no relationship between the detection rate and the tumor volume [7]. The EU-labeled *EWS/Fli-1* fusion mRNAs in MVs from TC135 cells were confirmed to be transferred into the other ES cells; however, the MVs were not transferred into non-ES HOS cells, suggesting a selectivity of transference. It was not easy for us to show a specific effect of the fusion mRNA in MVs on cell growth, because other genetic products such as mRNA, miRNA and protein were included in MVs from TC135 cells. Also, there may be tumor-suppressive molecules in addition to the *EWS/Fli-1* mRNAs among the other contents of the MVs. Recently, we found that MVs enhanced wound healing in recipient HUVEC cells (unpublished data), implying that MVs might influence tumor angiogenesis. The effects of MVs on recipient cells are still under intense investigation.

To our knowledge, this is the first report that the fusion mRNAs from a tumor-specific chromosome translocation can be detected within sarcoma cell-derived MVs isolated from the blood plasma of tumor bearing mice model. Recently, Miller et al., added Ewing sarcoma derived exosomes to healthy human blood plasma and they isolated the exosomes and detected the genetic information of Ewing sarcoma [28]. It is still necessary to determine whether the full-sized fusion mRNAs as observed in the cells are carried into the MVs and to clarify the effect of these MVs on the Ewing sarcoma cells. Nevertheless, on the basis of these results, we plan to examine the level of EWS/Fli-1 and EWS/ATF-1 fusion mRNAs in MVs in blood samples taken from patients and explore the sensitivity of the assay in a more clinical context. It is hoped that in the future, analysis of tumor-specific biomarkers within sarcoma derived MVs may assist the diagnosis of Ewing sarcoma patients and provide a convenient means of monitoring therapeutic approaches.

## Supporting Information

Figure S1 **We concentrated the MVs with the ExoMir kit.** Western blot analysis showed the expression of CD63, which is one of the markers of MVs.
(DOCX)

Movie S1 (MOV)
