# Supplementary material for: Ewing Sarcoma Cells Secrete EWS/Fli-1 Fusion mRNA via Microvesicles
Source: PLoS One. 2013 Oct 4;8(10):e77416. doi: 10.1371/journal.pone.0077416 (PMC3790721; doi:10.1371/journal.pone.0077416)

Figure S1

We concentrated the MVs with the ExoMir kit.

Western blot analysis showed the expression of CD63, which is one of the markers of MVs.

Cell: the protein from TC135 cell.

Pele: the protein from the pellet of cell culture medium after ultracentrifugation (100,000 rpm, 3 hours)

Top and Bot: the protein detected from top and bottom filters of ExoMir kit. The cell culture medium was filtered through the Exomir kit, as mentioned in “Isolation of extracellular microvesicles” section.


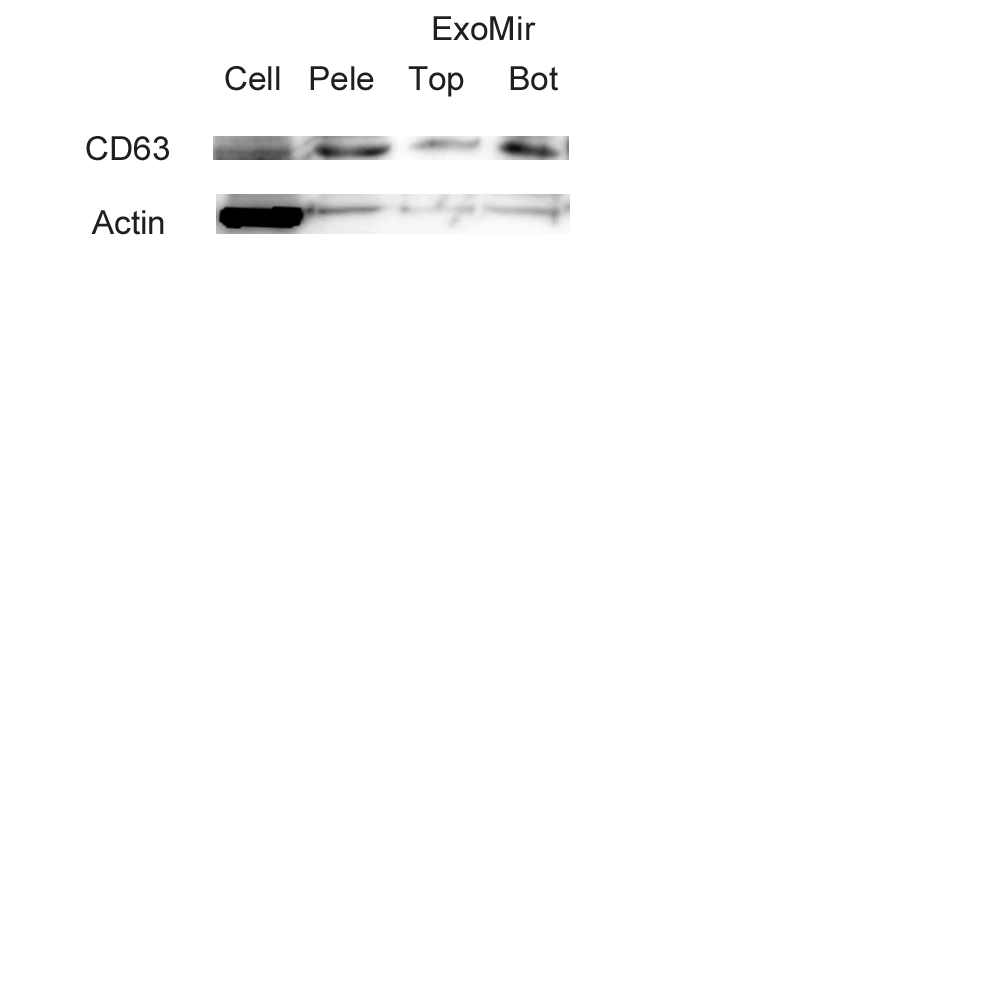

Supplement: Figure S1 — We concentrated the MVs with the ExoMir kit. Western blot analysis showed the expression of CD63, which is one of the markers of MVs. (DOCX) [file pone.0077416.s001.docx]
